# Supplementary material for: Orthogonal LoxPsym sites allow multiplexed site-specific recombination in prokaryotic and eukaryotic hosts
Source: Nat Commun. 2024 Feb 7;15:1113. doi: 10.1038/s41467-024-44996-8 (PMC10850332; doi:10.1038/s41467-024-44996-8)
Supplement: Supplementary file 1 — Supplementary Information File [file 41467_2024_44996_MOESM1_ESM.pdf]

Supplementary Information File

Orthogonal LoxPsym sites allow multiplexed site-specific recombination in prokaryotic and eukaryotic hosts

Charlotte Cautereels[1,2], Jolien Smets[1,2], Jonas De Saeger[3,4], Lloyd Cool[1,2,5], Yanmei Zhu[1,2], Anna Zimmermann[1,2], Jan Steensels[1,2], Anton Gorkovskiy[1,2], Thomas Jacobs[3,4] and Kevin J. Verstrepen[1,2]*

[1]VIB Laboratory for Systems Biology, VIB-KU Leuven Center for Microbiology, Leuven, 3001, Belgium

[2]CMPG Laboratory of Genetics and Genomics, Department M2S, KU Leuven, Leuven, 3001, Belgium

[3]Department of Plant Biotechnology and Bioinformatics, Ghent University, Technologiepark-Zwijnaarde 71, 9052 Ghent, Belgium

[4]VIB Center for Plant Systems Biology, Technologiepark-Zwijnaarde 71, 9052 Ghent, Belgium

[5]Laboratory of Socioecology and Social Evolution, KU Leuven, Leuven, Belgium

[*]Correspondence: kevin.verstrepen@kuleuven.be

This PDF file includes:

Supplemental Figures 1-10

Supplemental Tables 1-3

Supplemental Figures

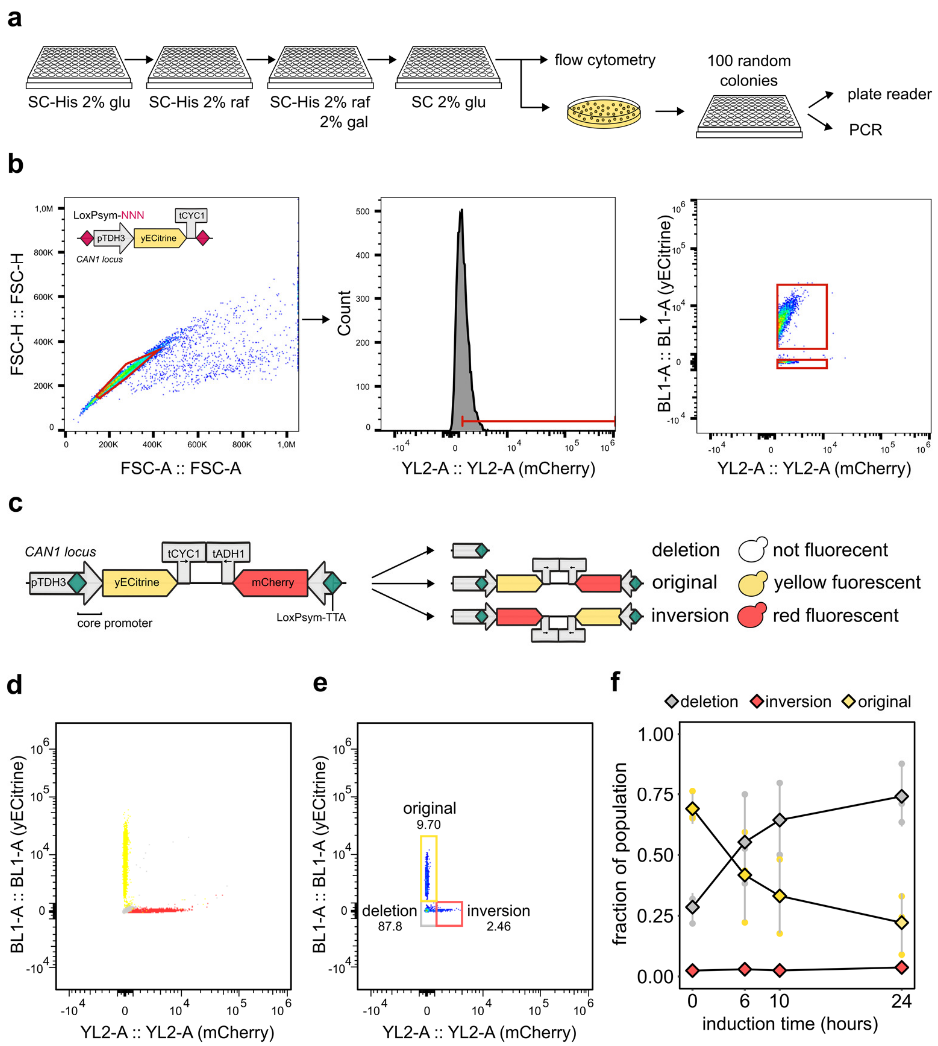

Supplemental Figure 1: Tracking recombination events in time reveals an increase of deletions caused by elongated induction time. a. Set-up of the fluorescent reporter assay. Cells were consecutively grown in SC-histidine medium (maintenance of the Cre-plasmid) with 2% glucose (glu), 2% raffinose (raf) and 2% raffinose 2% galactose (gal) for a specific induction time of the *Cre* recombinase expression, afterwards they were recovered in SC 2% glucose. Flow cytometry was next used to determine recombination efficiencies of the populations. To verify the assay, some populations were also plated, after which random colonies were picked for measurements with the plate reader and PCRs (see Supplemental Fig. 2). **b.** Three gating steps (red) were performed for during data analysis to distinguish cells that lost yECitrine fluorescence due to recombination (see Methods). **c.** Dual fluorescent reporter cassette integrated at the *CAN1* locus (left). LoxPsym-TTA flanks the reporter cassette so that a deletion, no recombination or an inversion of the surrounded construct results in a non-fluorescent, a yellow fluorescent or a red fluorescent strain respectively (right). **d.** yECitrine (BL 1-A) and mCherry (YL 2-A) fluorescence measured from three control strains with either a deletion (grey), inversion (red) or non-recombined construct (yellow), as schematically depicted in **c**. One population of three biological repeats is shown. **e.** Fluorescence measured from one population

(of three biological repeats) after 10 h of induction in SC-His 2% galactose 2% raffinose. Rectangular gates are defined based on the control populations shown in **c** and are used to define the percentage of the population that carried a deleted, inverted or original reporter construct. **f.** Fraction of the population carrying a deleted (grey), inverted (red) or original (yellow) reporter construct after 0, 6, 10 and 24 hours of induction of *Cre* recombinase expression by growth in galactose as shown in panel a. Data represents average of three biological replicates, error bars show the standard deviation. Source data for this figure are provided as a Source Data file.

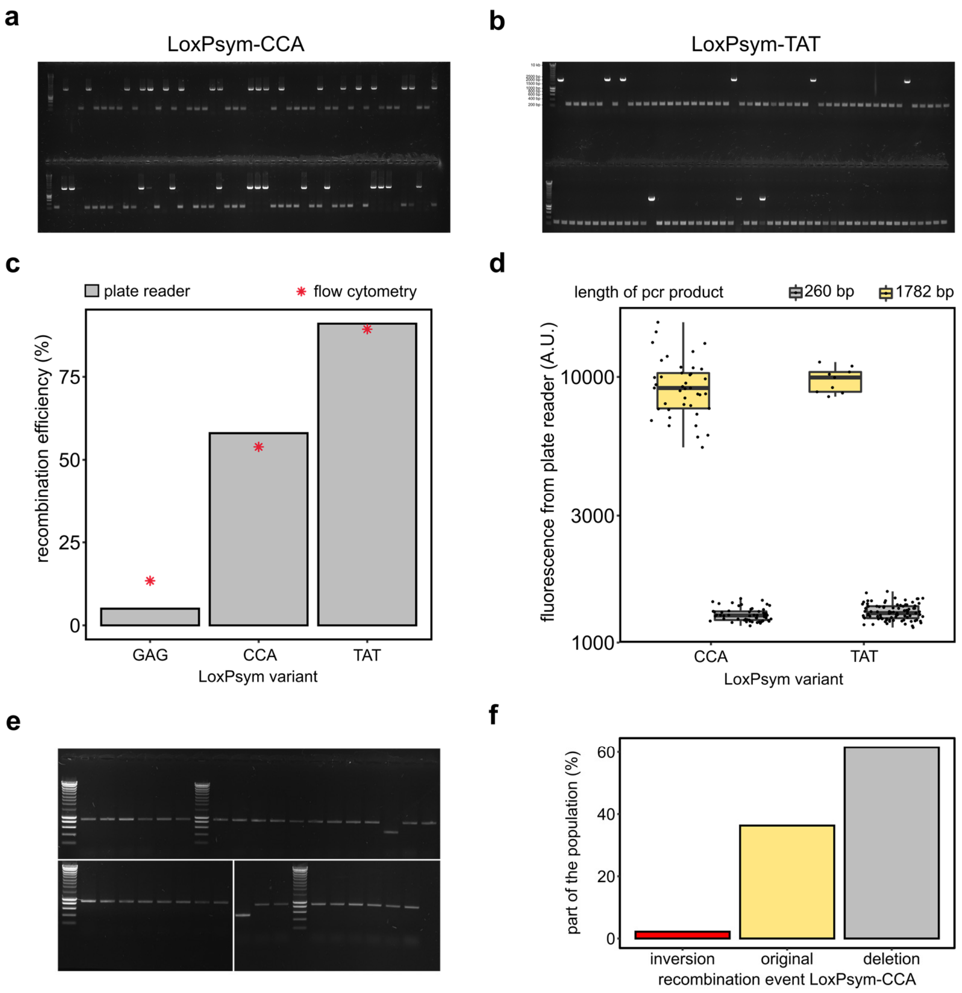

Supplemental Figure 2: PCR demonstrates that observed changes in fluorescence are linked to structural variation (recombination). a. PCR products amplified with primers outside of the LoxPsym-CCA-flanked fluorescence cassette, using the DNA from 100 randomly selected clones in which Cre recombination was induced for 6 h, after which cells were plated on YPD. Bands of 260 bp indicate a deletion of the reporter, bands of 1782 bp either indicate the original or an inversion of construct. Marker at both edges indicates 200 bp – 10 000 bp (SmartLadder MW-1700-10, Eurogentec). **b.** PCR similar to **a**, here for LoxPsym-TAT. **c.** Correspondence between recombination efficiencies obtained from 100 randomly selected single colonies (measured with the plate reader, grey bars) and from the population level (measured with the flow cytometer, red stars). **d.** Correspondence between the fluorescence level of single colonies on the y-axis (plate reader) and the genomic structural variation on the x-axis (PCR product length) of 100 randomly picked colonies from the population with LoxPsym-CCA and LoxPsym-TAT. The center line, box limits and whiskers of the boxplots indicate the median, first and third quartiles and 1.5 x interquartile range, respectively. **e.** PCR performed on the 35 clones with a long band in **a**, in which the junction between the LoxPsym-CCA flanked fluorescence cassette was amplified. Bands of 893 bp indicates the fluorescence cassette retained its original orientation, bands of 532 bp indicate the cassette was inverted. **f.** Percentage of clones (100 in total) with the original, deleted or inverted cassette flanked by LoxPsym-CCA. Source data for this figure are provided as a Source Data file.

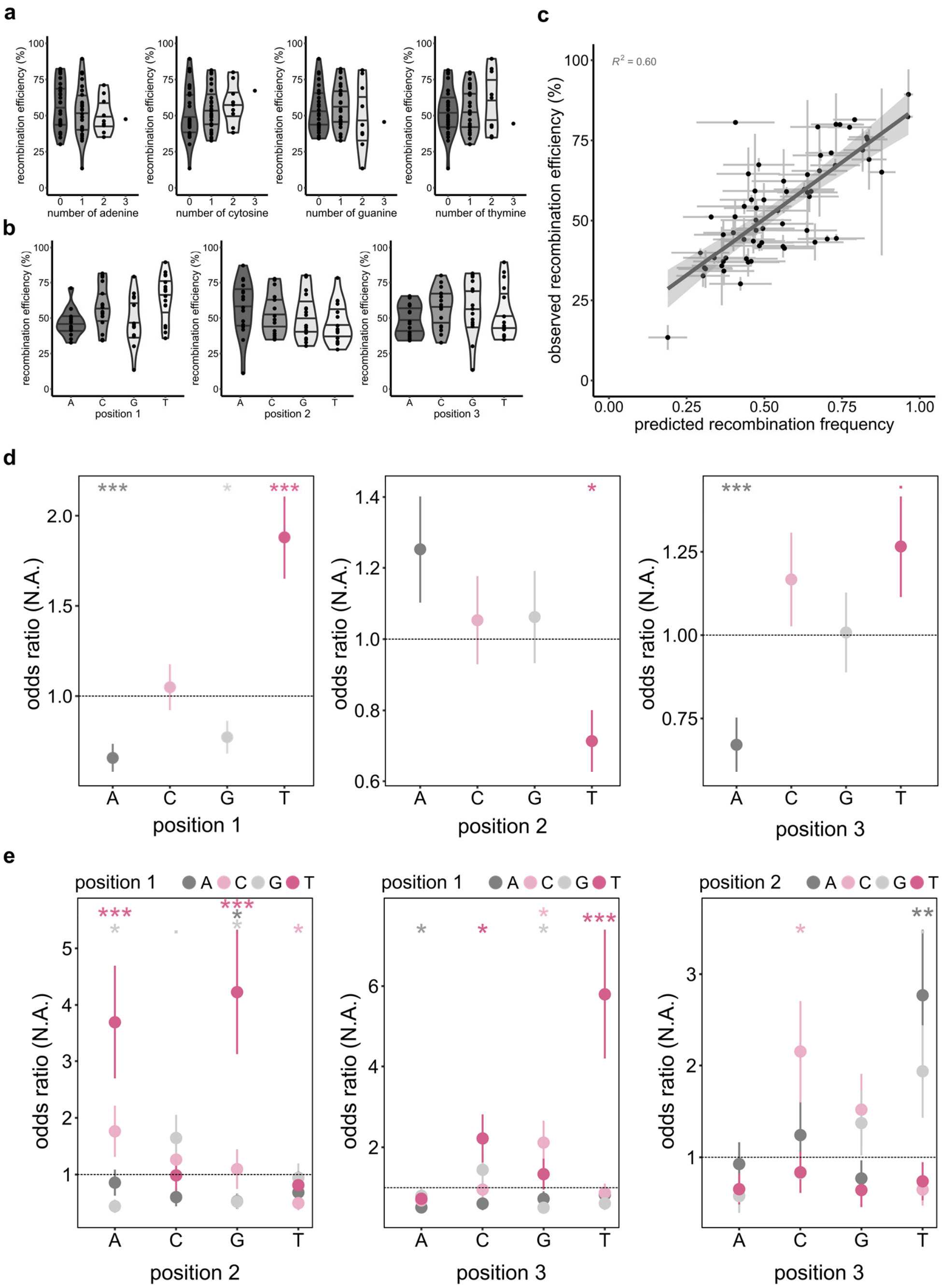

Supplemental Figure 3: Relationship between LoxPsym spacer sequence and recombination efficiency. a. Influence of the number of adenosine (A), cytosine (C), guanine (G) or thymine (T) in the spacer on the recombination efficiency. **b.** Influence of the position-effect of the nucleotides on the recombination efficiency. Horizontal lines in the violins represent the first quartile, median and third quartile. Dots represent the average recombination efficiencies of three biological repeats. **c.** Pearson correlation test between the average measured recombination efficiency (y-axis) and the efficiency predicted by generalized linear mixed-effects model fit 3 (x-axis) with R^2 = 0.60 and p = 5.3113e-14 (Pearson correlation test). Error bars represent standard error and deviation on the x- and y-axis, respectively. **d.** Magnification of **Fig. 1 f** showing the effects of single nucleotides on the recombination efficiency for each position of the spacer, calculated by the generalized linear mixed-effects model fit

3 (Supplemental Table 1). Odds ratios > 1 (dotted line) indicate that the event is more likely to occur as the predictor increases, odds ratios < 1 indicate the opposite. Dots and error bars represent the odds ratio and standard error, respectively. Statistics are performed on the log odds ratio scale and represent multiple pairwise-comparison by two-sided Tukey honest significant differences ('***' $p <$ 0.001, '**' $p < 0.01$, '*' $p < 0.05$, '.' $p < 0.1$). **e.** Magnification of **Fig. 1 g** showing the effects of the interactions between nucleotides at two positions of the LoxPsym spacer. Data and statistics similar to **d**. Source data for this figure are provided as a Source Data file.

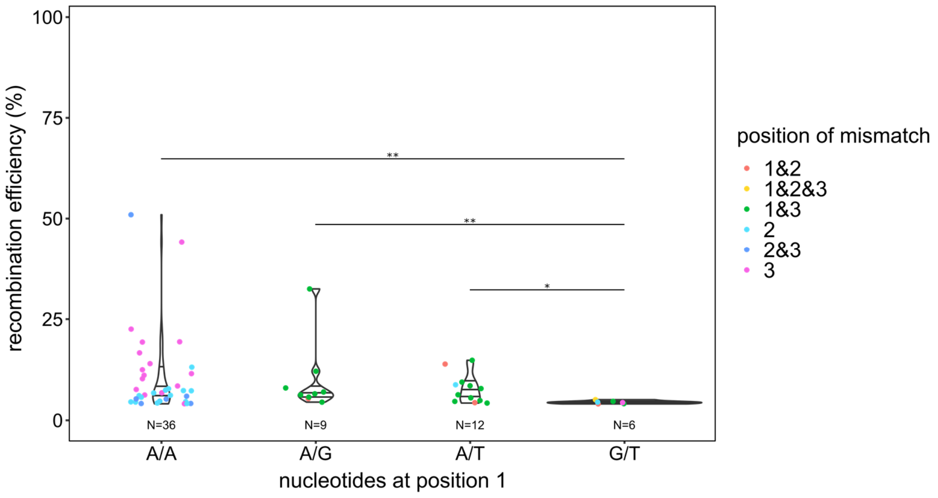

Supplemental Figure 4: Adenine at position 1 increases the degree of cross-reactivity between different LoxPsym variants. Spread of recombination efficiencies (y-axis) in function of the nucleotides at position 1 of the LoxPsym spacer sequence of the two cross-interacting LoxPsym variants (x-axis). Horizontal lines in the violins represent the first quartile, median and third quartile. Dots represent the average recombination efficiencies of three biological repeats. Color of the dots indicates the position(s) of the mismatch(es) between the interacting LoxPsym variants. Statistics by two-sided Wilcoxon rank sum test with p = 0.0084, 0.0084 and 0.0364 (from left to right). Source data for this figure are provided as a Source Data file.

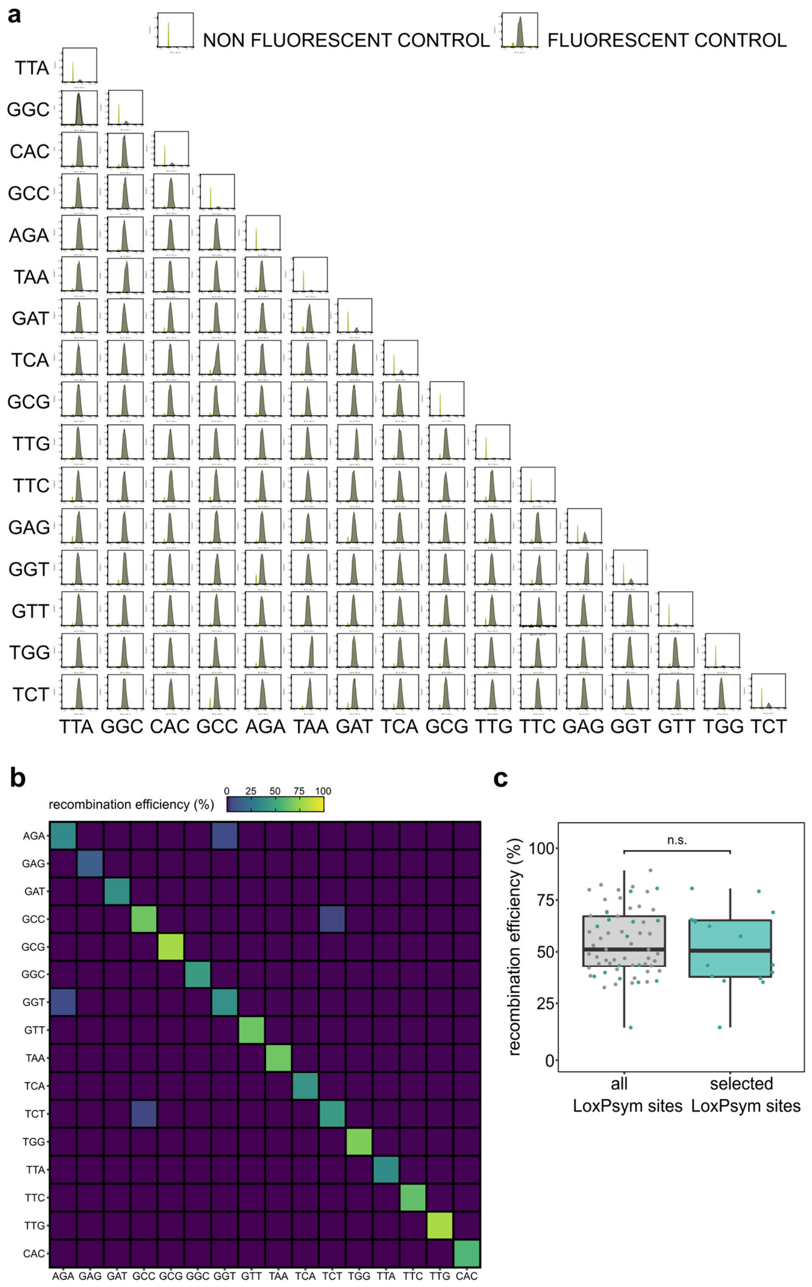

Supplemental Figure 5: The selection of 16 orthogonal LoxPsym variants for multiplex testing covers a wide range of recombination efficiencies. a. Flow cytometry data of the selected, orthogonal LoxPsym variants used for multiplexing (data for one of three biological replicates shown). LoxPsym variants are identified by the nucleotides at position 1, 2 & 3 at the bottom end left of the plots. Color of the histogram indicates cells were either counted as fluorescent (grey) or non-fluorescent (green). Gating of these groups was done based on the controls shown at the top. **b.** Interaction matrix representing the recombination efficiency (measured as the loss of fluorescence in the population) between different LoxPsym variants, identified by the nucleotides at position 1, 2 & 3 of the spacer. Data represent the average of at least 2 biological repeats. **c.** Range of recombination efficiencies (average of three biological repeats) of all 64 LoxPsym variants (grey) versus the group of LoxPsym

sites selected for multiplex testing (blue). Statistics by two-sided Fligner-Killeen test for comparison of the variance, with $p = 0.4206$. The center line, box limits and whiskers of the boxplots indicate the median, first and third quartiles and 1.5 x interquartile range, respectively. Source data for this figure are provided as a Source Data file.

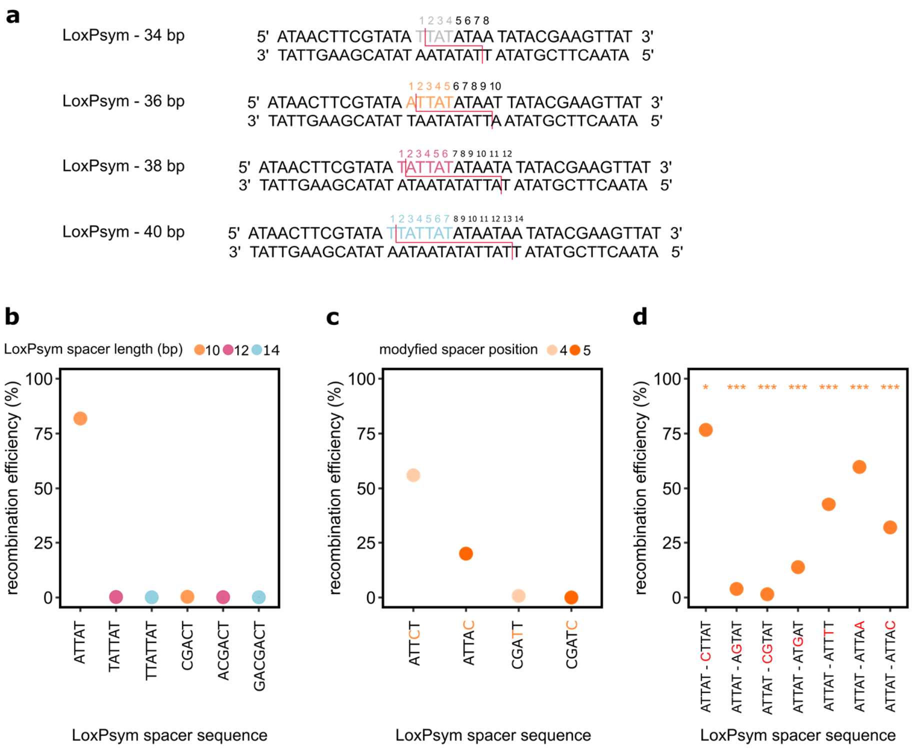

Supplemental Figure 6: LoxPsym variants with elongated spacers inhibit recombination depending on the spacer sequence. a. The LoxPsym sequence was elongated to 36, 38 or 40 bp, in the hypothesis to elongate the spacer to 10, 12 or 14 bp, respectively. The red line indicates the hypothetical positions where the recombinase might cause strand disruption. **b.** Recombination efficiency of elongated spacers, for which the first half of the sequences are displayed at the bottom. The length of the spacer was elongated to either 10 (orange), 12 (pink) or 14 (blue) nucleotides. Two different sequences were checked for each length. **c.** Effect of cytosine (C) at position 4 (light orange) or 5 (dark orange) on the recombination efficiency of the 10 bp spacers shown in **a**. **d.** Recombination efficiencies between LoxPsym variants with different 10 bp spacers. Positions of mismatches tested are 1, 2, 1&2, 3, 4 and 5. All data represent the mean of three biological replicates and error bars represent standard deviation. Statistics show multiple pairwise-comparison by Tukey honest significant differences to compare means against the mean of LoxPsym-ATTAT in **a**. Significance codes: '***' $p < 0.001$, '*' $p < 0.05$. Source data for this figure are provided as a Source Data file.

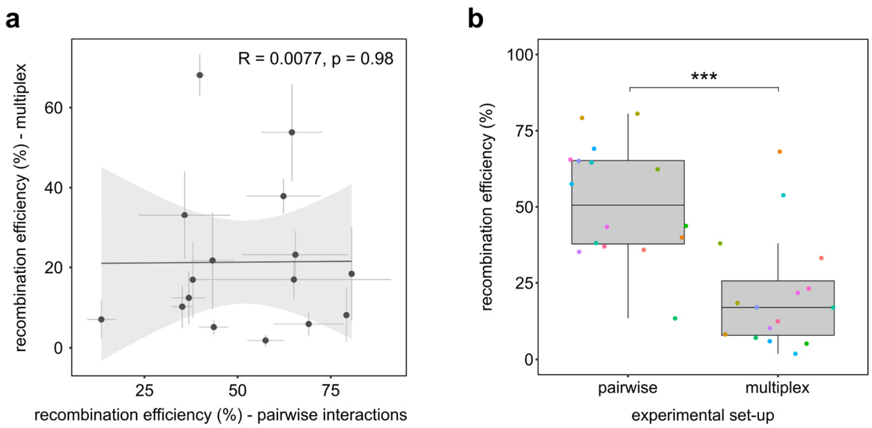

Supplemental Figure 7: Lower recombination efficiencies observed while multiplexing LoxPsym variants is correlated to the degree of insulation by other variants. a. Correlation between the recombination efficiencies (mean of three biological repeats) extracted from pairwise interactions using flow cytometry data (x-axis) and from multiplexing using colony color data. **b.** Range of recombination efficiencies (mean of three biological repeats) obtained from pairwise (left) and multiplex (right) experiments. The center line, box limits and whiskers of the boxplots indicate the median, first and third quartiles and 1.5 x interquartile range, respectively. Statistics by two-sided Wilcoxon test to compare the means, with p = 0.0001062. Source data for this figure are provided as a Source Data file.

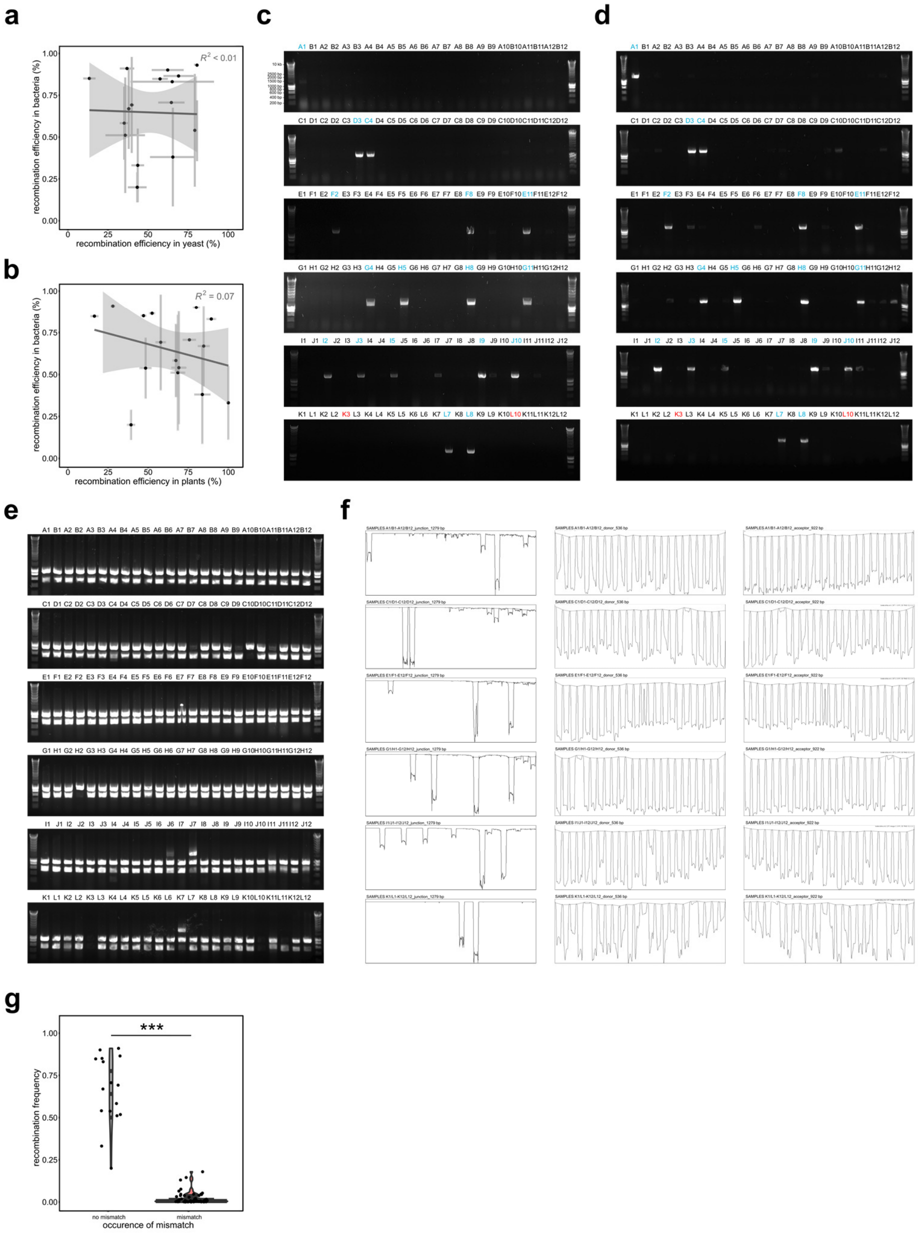

Supplemental Figure 8: PCR based approach for the determination of LoxPsym variant cross-reactivity in *E. coli*. a. Pearson correlation test between recombination efficiencies obtained from experiments in yeast (x-axis) and bacteria (y-axis), $p = 0.9116$. Data represents the average of 3 biological repeats (yeast) and 3 biological repeats performed in technical duplicate (bacteria). Error bars indicate standard deviation. **b.** Pearson correlation test between recombination efficiencies obtained from experiments in plants (x-axis) and bacteria (y-axis), $p = 0.3010$. Data represents the average of 2 biological repeats performed in technical triplicate (plants) and 3 biological repeats performed in technical duplicate (bacteria). Error bars indicate standard deviation. **c.** Amplification of the junction between recombined donor and acceptor plasmids (with oligonucleotides OF/R44). PCR was performed on a mixture of templates derived from three independent biological repeats. Amplicons of 1279 bp indicate recombination between plasmids was successful. Marker at both edges indicates 200 bp – 10 000 bp (SmartLadder MW-1700-10, Eurogentec). Codes on top of the gel images indicate the sample used for LoxPsym cross-reactivity testing, more information provided in

Supplementary Data 1, cross yeast. Blue wells indicate identical LoxPsym sites were present in donor and acceptor plasmids and presence of the amplicon was expected, red wells indicate control samples for which no band was expected. **d.** Technical replicate of the result shown in **c**. **e.** Amplification of the donor (536 bp, with oligonucleotides OF/R45) and acceptor (922 bp, with oligonucleotides OF/R46) plasmids were performed separately and simultaneously loaded on the gel. **f.** Densitometric analysis of the gel pictures depicted in panel **c** (left) and **e** (middle and right). Peak areas of the junction were normalized by areas of the control amplicons, which was used to present the frequencies of recombination for each sample in **Fig. 4**. **g.** Violins indicate recombination frequency calculated from densitometric analysis, grouped by the absence (grey) of presence (red) of a mismatch between recombining LoxPsym variants in *E. coli.* Horizontal lines in the violins represent the first quartile, median and third quartile. Statistics by two-sided Kruskal-Wallis test with p = 1.111e-09. Source data for this figure are provided as a Source Data file.

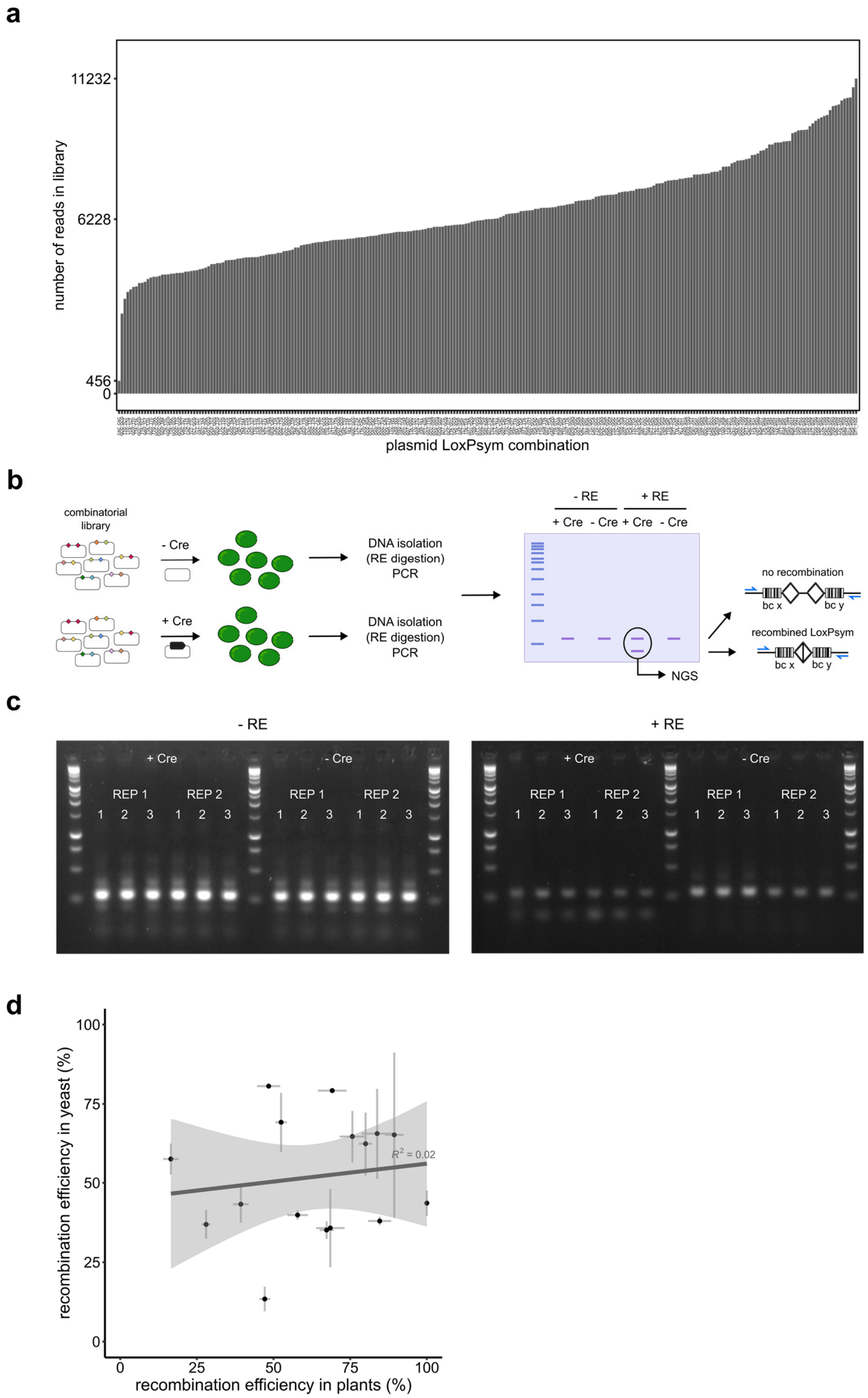

Supplemental Figure 9: Determination of LoxPsym variant cross-reactivity in *Z. mays*. a. Number of reads represent the abundance of each plasmid in the combinatorial library, obtained by NGS of the library input before transfection. These read counts were used for normalization of the counts obtained for the recombined LoxPsym amplicons. **b.** Experimental workflow for determination of cross-reactivity between LoxPsym variants. Maize protoplast cells were transfected with the combinatorial library of plasmids carrying all 256 combinations of LoxPsym variants on separate plasmids. Library was co-transfected with either a plasmid encoding the Cre recombinase or an empty vector. Consecutively, DNA isolation (, restriction enzyme (RE) digestion to destroy non-recombined

plasmids) and PCR were performed. Gel electrophoresis allowed to separate fragments with recombined LoxPsym sites from those without the presence of recombination and the reaction of samples indicated with RE+ Cre+ were send for next generation sequencing (NGS). The barcodes were used to identify which two LoxPsym variants recombined together. **c.** Agarose gel with separated DNA amplicons resulting from PCR on DNA isolated from protoplasts, that were either co-transfected with either Cre or empty plasmid (-RE, left). Prior to amplification, the template DNA was first treated with restriction enzymes (+RE) to cut the linker between non-recombined LoxPsym variants (right). **d.** Pearson correlation test between recombination efficiencies obtained from experiments in plants (x-axis) and yeast (y-axis), $p = 0.6056$. Data represents the average of 2 biological repeats performed in technical triplicate (plants) and 3 biological repeats (yeast). Error bars indicate standard deviation. Source data for this figure are provided as a Source Data file.

```
# Make empty list to read list from a file
data = []

# Open file containing the NGS reads and read the content in a list. Here
the example is given with BIO1_R1.txt. The NGS reads contain a mix of
recombined and native plasmid species. When counting the reads from the
protoplast experiment, only the short species need to be counted. For
counting the input library, the if function in the loop is set with the
values 266 and 270.
with open('BIO1_R1.txt', 'r') as fp:
    for line in fp:
        if 128 <= len(line) <= 132:
        # Remove linebreak from a current name
        # Linebreak is the last character of each line
            x = line[:-1]

        # Add current item to the list
            data.append(x)

# Display list
print(data)

# Make empty list to read list from a file
bar = []

# Open the file containing the possible barcode combinations and read the
content in a list. The barcodeCombo.txt file contains all 256 possible
barcode combinations separated by ".*"
with open('barcodeCombo.txt', 'r') as L:
    for line in L:
        # Remove linebreak from a current name
        # Linebreak is the last character of each line
        x = line[:-1]

        # Add current item to the list
        bar.append(x)

# Display barcode list
print(bar)


# Count number of reads that contain a given barcode combination and list
all counts

import re

for i in bar:
    p = re.compile(i)
    a=sum(1 for x in data if p.match(x))
    print(a)
```

Supplemental Figure 10: Phyton script for NGS analysis.

Supplemental Tables

Supplemental Table 1: Generalized linear mixed-effects models (GLME) predicting recombination efficiency from LoxPsym spacer sequence.

Fit	Generalized linear mixed-effects model[1]	Df	AICc
fit1	glmer(efficiency~pos1*pos2*pos3+(1|rep),family=binomial, weight=cellcount,data=data)	64	3207.405
fit2	glmer(efficiency~pos1+pos2+pos3+(1|rep),family=binomial, weight=cellcount,data=data)	11	3182.601
fit3	glmer(efficiency~pos1+pos2+pos3+pos1:pos2+pos2:pos3+pos1:pos3+ (1|rep),family=binomial,weight=cellcount,data=data)	38	3165.110
fit3.1	glmer(efficiency~pos2+pos3+pos1:pos2+pos2:pos3+pos1:pos3+(1|rep), family=binomial,weight=cellcount,data=data)	38	3165.110
fit3.2	glmer(efficiency~pos1+pos3+pos1:pos2+pos2:pos3+pos1:pos3+(1|rep), family=binomial,weight=cellcount,data=data)	38	3165.110
fit3.3	glmer(efficiency~pos1+pos2+pos1:pos2+pos2:pos3+pos1:pos3+(1|rep), family=binomial,weight=cellcount,data=data)	38	3165.110
fit3.4	glmer(efficiency~pos1+pos2+pos3+pos2:pos3+pos1:pos3+(1|rep), family=binomial,weight=cellcount,data=data)	29	3180.897
fit3.5	glmer(efficiency~pos1+pos2+pos3+pos1:pos2+pos1:pos3+(1|rep), family=binomial,weight=cellcount,data=data)	29	3165.127
fit3.6	glmer(efficiency~pos1+pos2+pos3+pos1:pos2+pos2:pos3+(1|rep), family=binomial,weight=cellcount,data=data)	29	3172.445

[1] Nine GLMEs (fit1-fit3.6) were constructed to model the observed recombination efficiency ('efficiency') in function of the nucleotides at position 1 ('pos1'), 2 ('pos2') and 3 ('pos3') of the spacer. The population size was used as weights ('cellcount'). The biological replicates ('rep') were included as a random effect

Supplemental Table 2: Barcodes associated with LoxPsym variants in the combinatorial LoxPsym library (*Z. mays*).

LoxPsym-NNN	barcode (8 bp)
TTA	ACACAGGC
GGC	ACTGTTAG
CAC	ATAGAGTC
GCC	ATTAGCTG
AGA	CAGTTCCA
TAA	CCGTATAT
GAT	CGCAAGCT
TCA	CTGGCACA
GCG	GACACTAA
TTG	GGAGTAGA
TTC	GGCTCTTG
GAG	GGGAGATC
GGT	TACTGCAG
GTT	TATCCAGT
TGG	TCTCGCCT
TCT	TTACTGGC

Supplemental Table 3: Golden Gate cloning scheme for construction of plasmids used in *Z. mays*.

plasmid nb	plasmid name	backbone	part A	part B	part C	part D	part E	part F
P51-P216	LoxPsym combinatorial library	pGG-AG-KmR (Vector ID: 14_32; https://gatewayvectors.vib.be/)	OF/R47	LoxPsym variant (OF/R50-OF/R65)	Linker (OF/R49)	LoxPsym variant (OF/R66-OF/R81)	OF/R48	-
P218	pUBI-Cre-NOST	p35S-mCherry-NLS_CmR-ccdB (Vector ID: 18_17; https://gatewayvectors.vib.be/)	A-ZmUBI-B (fragment from pGG-A-pZmUBIL-B, Vector ID: 6_55)	B-linker-C (Lampropoulos et al., 2013)	C-MoCre-D (fragment from pGG-C-MoCRE*-D, Vector ID: 19_47)	D-linker-E (Lampropoulos et al., 2013)	E-NOST-F (fragment from pGG-E-NOST-F, Vector ID: 6_68)	F-linkerII-G (fragment from pGG-F-linkerII-G, Vector ID: 8_38)
