# Supplementary material for: Orthogonal LoxPsym sites allow multiplexed site-specific recombination in prokaryotic and eukaryotic hosts
Source: Nat Commun. 2024 Feb 7;15:1113. doi: 10.1038/s41467-024-44996-8 (PMC10850332; doi:10.1038/s41467-024-44996-8)
Supplement: Supplementary file 2 — Reporting Summary [file 41467_2024_44996_MOESM2_ESM.pdf]

nature portfolio

Corresponding author(s): Verstrepen Kevin

Last updated by author(s): Jan 4, 2024

Reporting Summary

Nature Portfolio wishes to improve the reproducibility of the work that we publish. This form provides structure for consistency and transparency in reporting. For further information on Nature Portfolio policies, see our Editorial Policies and the Editorial Policy Checklist.

Statistics

For all statistical analyses, confirm that the following items are present in the figure legend, table legend, main text, or Methods section.

n/a	Confirmed	
☐	☒	The exact sample size (*n*) for each experimental group/condition, given as a discrete number and unit of measurement
☐	☒	A statement on whether measurements were taken from distinct samples or whether the same sample was measured repeatedly
☐	☒	The statistical test(s) used AND whether they are one- or two-sided<br>*Only common tests should be described solely by name; describe more complex techniques in the Methods section.*
☐	☒	A description of all covariates tested
☐	☒	A description of any assumptions or corrections, such as tests of normality and adjustment for multiple comparisons
☐	☒	A full description of the statistical parameters including central tendency (e.g. means) or other basic estimates (e.g. regression coefficient) AND variation (e.g. standard deviation) or associated estimates of uncertainty (e.g. confidence intervals)
☐	☒	For null hypothesis testing, the test statistic (e.g. *F*, *t*, *r*) with confidence intervals, effect sizes, degrees of freedom and *P* value noted<br>*Give P values as exact values whenever suitable.*
☒	☐	For Bayesian analysis, information on the choice of priors and Markov chain Monte Carlo settings
☒	☐	For hierarchical and complex designs, identification of the appropriate level for tests and full reporting of outcomes
☒	☐	Estimates of effect sizes (e.g. Cohen's *d*, Pearson's *r*), indicating how they were calculated

Our web collection on statistics for biologists contains articles on many of the points above.

Software and code

Policy information about availability of computer code

Data collection	No software was used for data collection.
Data analysis	All software used for data analysis is described in the manuscript. More specifically,<br>- fluorescence analysis: FlowJo version 10.6.2<br>- model development and statistical analysis: R studio version 4.3.0 using R package lme4, emeans, effects<br>- analysis of amplicons using capillary electrophoresis: QIAxcel ScreenGel version 1.1.0<br>- analysis of NGS data: Geneious Prime version 2022.2.1 and Phyton version 3.9<br>- analysis of Sanger sequencing data: SnapGene version 5.2.4

For manuscripts utilizing custom algorithms or software that are central to the research but not yet described in published literature, software must be made available to editors and reviewers. We strongly encourage code deposition in a community repository (e.g. GitHub). See the Nature Portfolio guidelines for submitting code & software for further information.

Data

Policy information about availability of data

All manuscripts must include a data availability statement. This statement should provide the following information, where applicable:

- Accession codes, unique identifiers, or web links for publicly available datasets
- A description of any restrictions on data availability
- For clinical datasets or third party data, please ensure that the statement adheres to our policy

All data supporting the findings of this study are available within the paper and its supplementary information files. NGS and Sanger sequencing data have been deposited to NCBI Sequence Read Archive database under accession code PRJNA1002847.

Research involving human participants, their data, or biological material

Policy information about studies with human participants or human data. See also policy information about sex, gender (identity/presentation), and sexual orientation and race, ethnicity and racism.

Reporting on sex and gender	No information on sex and gender has been collected.
Reporting on race, ethnicity, or other socially relevant groupings	No information on race, ethnicity, or other socially relevant groupings has been collected.
Population characteristics	No human research participants have been used.
Recruitment	No participants have been recruited.
Ethics oversight	No research involving human participants, their data, or biological material has been performed.

Note that full information on the approval of the study protocol must also be provided in the manuscript.

Field-specific reporting

Please select the one below that is the best fit for your research. If you are not sure, read the appropriate sections before making your selection.

☒ Life sciences ☐ Behavioural & social sciences ☐ Ecological, evolutionary & environmental sciences

For a reference copy of the document with all sections, see nature.com/documents/nr-reporting-summary-flat.pdf

Life sciences study design

All studies must disclose on these points even when the disclosure is negative.

Sample size	No sample size calculation was performed; sample size was determined based on previous studies. Sample sizes in flow cytometry were minimally 10, 000 (with three biological replicates, unless indicated otherwise in the Source Data file). Sample sizes for colony counting were minimally 13 (with six biological replicates, unless indicated otherwise in the Source Data file). Experiments involving bacteria and plants used minimally 3 biological repeats and 2 biological repeats (with three technical replicates), respectively. All sample sizes were chosen to allow for statistical analysis and to ensure reproducibility.
Data exclusions	No data was excluded.
Replication	To verify reproducibility of experiments, experiments were performed using biological (n>=2) and technical (n>=2) replicates, for which variability of the data is provided by standard deviation. No replicates were excluded from the data provided in the manuscript.
Randomization	Samples were randomly allocated into experimental groups. Control/reference samples were included in each group.
Blinding	The investigators were blinded to group allocation during data collection and analysis (determination of recombination efficiencies) of all data presented in the paper.

Reporting for specific materials, systems and methods

We require information from authors about some types of materials, experimental systems and methods used in many studies. Here, indicate whether each material, system or method listed is relevant to your study. If you are not sure if a list item applies to your research, read the appropriate section before selecting a response.

Materials & experimental systems

n/a	Involved in the study	
x		Antibodies
x		Eukaryotic cell lines
x		Palaeontology and archaeology
x		Animals and other organisms
x		Clinical data
x		Dual use research of concern
	x	Plants

Methods

n/a	Involved in the study	
x		ChIP-seq
	x	Flow cytometry
x		MRI-based neuroimaging

Dual use research of concern

Policy information about dual use research of concern

Hazards

Could the accidental, deliberate or reckless misuse of agents or technologies generated in the work, or the application of information presented in the manuscript, pose a threat to:

No	Yes	
x		Public health
x		National security
x		Crops and/or livestock
x		Ecosystems
x		Any other significant area

Experiments of concern

Does the work involve any of these experiments of concern:

No	Yes	
x		Demonstrate how to render a vaccine ineffective
x		Confer resistance to therapeutically useful antibiotics or antiviral agents
x		Enhance the virulence of a pathogen or render a nonpathogen virulent
x		Increase transmissibility of a pathogen
x		Alter the host range of a pathogen
x		Enable evasion of diagnostic/detection modalities
x		Enable the weaponization of a biological agent or toxin
x		Any other potentially harmful combination of experiments and agents

Flow Cytometry

Plots

Confirm that:

- [x] The axis labels state the marker and fluorochrome used (e.g. CD4-FITC).
- [x] The axis scales are clearly visible. Include numbers along axes only for bottom left plot of group (a 'group' is an analysis of identical markers).
- [x] All plots are contour plots with outliers or pseudocolor plots.
- [x] A numerical value for number of cells or percentage (with statistics) is provided.

Methodology

Sample preparation	Samples preparation is described in the methods section. Cell cultures were diluted in focusing fluid to obtain a detection rate of approximately 500-5000 events/second.
Instrument	Attune NxT Flow Cytometer and Auto Sampler

Software	FlowJo version 10.6.2
Cell population abundance	No post-sorted cell populations were used.
Gating strategy	Several gating steps were performed consecutively: FSC-H/FSC-A (single cells); YL2-A + (mCherry used as a positive control, baseline set based on a fluorescent and non-fluorescent control strain); BL1A + or BL1-A - (yECitrine used as a read out of recombination efficiency, baseline set based on a fluorescent and non-fluorescent control strain)

☒ Tick this box to confirm that a figure exemplifying the gating strategy is provided in the Supplementary Information.
