# Supplementary material for: Orthogonal LoxPsym sites allow multiplexed site-specific recombination in prokaryotic and eukaryotic hosts
Source: Nat Commun. 2024 Feb 7;15:1113. doi: 10.1038/s41467-024-44996-8 (PMC10850332; doi:10.1038/s41467-024-44996-8)
Supplement: Supplementary file 3 — Description of Additional Supplementary Files [file 41467_2024_44996_MOESM3_ESM.pdf]

Title: Supplementary Data 1
Description: LoxPsym, GLME, counts, cross yeast, densitometry, cross bacteria, NGS, plasmids, oligo's, constructs
